# Signatures of a magnetic field-induced unconventional nematic liquid in the frustrated and anisotropic spin-chain cuprate LiCuSbO_4_

**DOI:** 10.1038/s41598-017-06525-0

**Published:** 2017-07-27

**Authors:** H.-J. Grafe, S. Nishimoto, M. Iakovleva, E. Vavilova, L. Spillecke, A. Alfonsov, M.-I. Sturza, S. Wurmehl, H. Nojiri, H. Rosner, J. Richter, U. K. Rößler, S.-L. Drechsler, V. Kataev, B. Büchner

**Affiliations:** 1 Leibniz Institute for Solid State and Materials Research IFW-Dresden, D-01171 Dresden, Germany; 2 Institute for Theoretical Physics, Technical University Dresden, D-01069 Dresden, Germany; 3 Zavoisky Physical-Technical Institute of the Russian Academy of Sciences, 420029 Kazan, Russia; 4 Institute for Solid State Physics, Technical University Dresden, D-01069 Dresden, Germany; 5 Institute of Materials Research, Tohoku University, 980-8577 Sendai, Japan; 6 Max-Planck-Institute for Chemical Physics of Solids, Dresden, Germany; 7 Universität Magdeburg, Institut für Theoretische Physik, Magdeburg, Germany

## Abstract

Modern theories of quantum magnetism predict exotic multipolar states in weakly interacting strongly frustrated spin-1/2 Heisenberg chains with ferromagnetic nearest neighbor (NN) inchain exchange in high magnetic fields. Experimentally these states remained elusive so far. Here we report strong indications of a magnetic field-induced nematic liquid arising above a field of ~13 T in the edge-sharing chain cuprate LiSbCuO_4_ ≡ LiCuSbO_4_. This interpretation is based on the observation of a field induced spin-gap in the measurements of the ^7^Li NMR spin relaxation rate *T*_1_^−1^ as well as a contrasting field-dependent power-law behavior of *T*_1_^−1^ vs. *T* and is further supported by static magnetization and ESR data. An underlying theoretical microscopic approach favoring a nematic scenario is based essentially on the NN XYZ exchange anisotropy within a model for frustrated spin-1/2 chains and is investigated by the DMRG technique. The employed exchange parameters are justified qualitatively by electronic structure calculations for LiCuSbO_4_.

## Introduction

Strong electronic correlations in solids may give rise to novel ground states of matter such as electronic liquid crystal phases in correlated metals or spin liquid states in insulating quantum magnets^1, 2^. Theory predicts that in the latter systems conventional long-range magnetic dipolar order can be suppressed down to *T* = 0 due to frustration of magnetic interactions and/or quantum fluctuations (for a review see, e.g., ref. 3). Though individual spins remain non-ordered in the spin liquid, higher rank magnetic multipoles (quadrupoles, octupoles etc.) can order under favorable conditions^4^. Such an exotic multipolar (MP) order does not break the time-reversal symmetry and is often referred to as a “hidden order” since it is difficult to detect experimentally by most of the available techniques sensitive to magnetic dipole moments, only. However, the spin rotational symmetry is broken in this hidden phase which is therefore also called a spin-nematic state in the simplest quadropolar case, in analogy with the nematic order in liquid crystals, where the translational crystalline order is absent but the rotational symmetry is broken.

A prominent example of a spin liquid is a single Heisenberg spin-1/2 chain with the nearest neighbor (NN) antiferromagnetic (AFM) interaction whose ground state is described by the so-called gapless Tomonaga-Luttinger (TL) spin liquid^5^. Further complexity can be brought in the problem by including an AFM next-nearest neighbor (NNN) interaction *J*_2_. Irrespective of the sign of the NN coupling *J*_1_, AFM *J*_2_ causes spin frustration and may yield different phases depending on the frustration ratio *α* =  |*J*_2_/*J*_1_|^6–8^. In particular, pioneering theoretical works^8–16^ devoted mainly to one-dimensional (1D) isotropic frustrated *J*_1_(FM) − *J*_2_(AFM) chain models have predicted unusual field-induced MP states near the saturation field *H*_sat_ above which at *T* = 0 all spins are aligned by an external magnetic field *H*. These states form a TL-liquid of multiple *p*-bound states of magnons corresponding to nematic, triatic, quartic MP phases ($p=2,3,4,\ldots$). For interacting chains within 2D or 3D arrangements, the MP phases strongly compete with collinear longitudinal *H*-dependent incommensurate spin density wave (SDW_*p*_) phases predominant at lower fields^17^. (Here the index *p* indicates the neighboring MP state.) Albeit MP phases might coexist with *non*-*collinear* strongly fluctuating dipolar states^18^.

**Figure 1:**
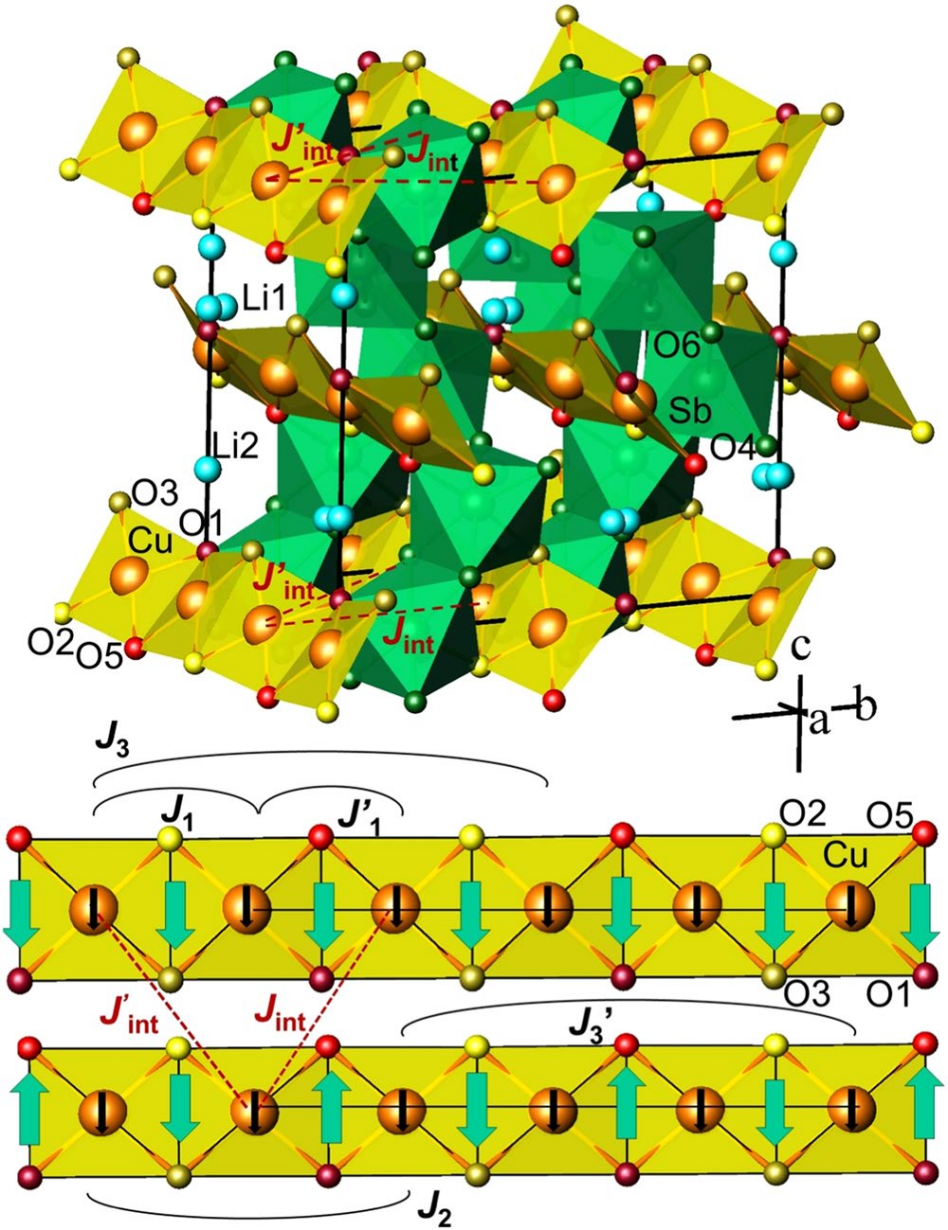
Top: crystallographic structure of LiSbCuO_4_ ≡ LiCuSbO_4_. Cu^2+^ ions (orange) are bonded covalently to four *nonequivalent* O^2−^ ligands and form buckled non-planar CuO_4_ plaquettes. Edge-shared CuO_4_ plaquettes form non-ideal alternatingly tilted stacks (along the *c*-axis) of CuO_2_ *chains* (colored yellow and brown, respectively) running along the *a*-axis. The chains are interconnected with SbO_6_ octahedra shown in blue-green. The Li^+^ ions (bright blue balls) occupy two positions. The split Li1 position is shown with overlapping balls. Bottom: Schematic view of two neighboring individual CuO_2_ chains within the *ab*-plane ignoring their tilting and buckling (cf. Top). The relevant intrachain exchange paths are indicated by black arcs. The four nonequivalent O^2−^ ions within a CuO_4_-plaquette give rise to different left and right Cu-Cu bonds along the *a*-axis causing this way an *alternated* $({J}_{1},{J}_{1}^{^{\prime} })$ − *J*_2_ spin chain with small nonequivalent third neighbor couplings $({J}_{3},{J}_{3}^{^{\prime} })$. Black arrows denote the magnetically active spins and bright blue arrows the DM vectors that are confined to the (*bc*)-plane ⊥ to the chain axis whereas their mutual orientation can be arbitrary. For illustration the two limiting configurations, uniform and staggered are depicted that have been studied theoretically in single-chain approximation. Dark dashed line: the frustrated weak interchain couplings *J*_*int*_ and ${J}_{int}^{^{\prime} }\sim 1\,{\rm{K}}\ll {J}_{2} < |{J}_{1}|$, $|{J}_{1}^{^{\prime} }|$ in the basal (*ab*)-plane (see also Top).

Low-D spin networks can be found in 3D transition metal (TM) oxides. A specific geometry of the chemical bonds can yield chains of TM ions magnetically coupled mainly via oxygen ligands in one direction. A sizable and still increasing number of frustrated CuO_2_ spin-1/2 chain compounds is currently available. Nevertheless, the very existence of an MP state is not yet proved. Also the possibility of a coexistence of an MP state (and even of an ordered phase) with some other dipolar magnetic phases or corresponding strong fluctuations of them remains unclear up to now. The quest for MP phases focussed in the last years mainly on two compounds, namely LiCuVO_4_^19–21^ and PbCuSO_4_(OH)_2_ (linarite)^22^. Both materials exhibit at ambient fields a 3D-non-collinear spiral type dipolar order at low *T* due to residual interchain couplings. Various field-induced collinear SDW_*p*_ phases were detected but the theoretically proposed neighboring MP phases at higher fields remain elusive. In particular, in the case of LiCuVO_4_ it was concluded that the spin-nematic phase, if it exists at all, could be established only in a very narrow field range of 1 T below ${\mu }_{0}{H}_{{\rm{sat}}}\approx 41.4\,\,{\rm{T}}$ ^20^. For completeness we note, that a suggested “bond-nematic” phase with a coexisting collinear SDW_2_-magnetic structure and nematic fluctuations proposed in ref. 23 has been arguably questioned in ref. 17 based on the analysis of an isotropic quasi-2D field-theory model for interacting chains. The very broad phase below μ_0_ *H*_sat_ ~ 43 T was ascribed instead to the mentioned above SDW_2_-phase, only. Indeed, from the theoretical side it has become clear that the interchain coupling that causes dipolar magnetic order can easily destroy fragile MP phases whereas easy-axis exchange anisotropy may stabilize them^24, 25^. In this context the recent synthesis and the first physical study of a novel member of the frustrated CuO_2_-chain family, the strongly frustrated *J*_1_(FM) − *J*_2_(AFM) quasi-1D compound LiCuSbO_4_ (Fig. 1), is noteworthy^26^. It exhibits short-range incommensurate spin correlations below *T* ~ 9 K but, in contrast to the spiral spin-chain compounds LiCuVO_4_ and LiCuZrO_4_^27, 28^, does not show long-range magnetic order at *H* = 0 down to *T* ~ 0.1 K. This points to a very weak or specific interchain coupling in LiCuSbO_4_. Moreover, a sizeable exchange anisotropy was estimated here, too.

Here we report the results of ^7^Li NMR relaxation rate ${T}_{1}^{-1}$ measurements in LiCuSbO_4_ in a broad field range of 3–16 T. A surprising contrasting behavior of ${T}_{1}^{-1}$ vs. *T* is observed around a special crossover field ${\mu }_{0}{H}_{{\rm{c}}1}\approx 13{\rm{T}}$ within a rather narrow field range. At *H* < *H*_c1_, ${T}_{1}^{-1}$ (*T*) exhibits a diverging power-law behavior suggesting a static magnetic order at lower temperatures. In contrast, in higher magnetic fields *H* > *H*_c1_, ${T}_{1}^{-1}$ (*T*) turns into an exponential decrease indicative of the opening of a spin gap above the narrow crossover high-field region. Our analysis of the static magnetization and ESR data rules out saturation of the magnetization or Dzyaloshinskii-Moriya (DM) couplings as possible conventional reasons for the opening of this spin gap. We argue that this gap should be considered as one of the signatures of a distinctive but nevertheless naturally “hidden” for a dipolar sensitive probe MP state. In particular, we argue that such a peculiar behavior of ${T}_{1}^{-1}$ is due to the occurrence of a dipolar spin-liquid state confined to a certain field range below *H*_c1_ which crosses over to the competing anisotropic spin-nematic liquid state which is stabilized above *H*_c1_. Our theoretical analysis employing the density matrix renormalization group (DMRG) technique indeed reveals a broad stability region of an unconventional spin-nematic liquid state in LiCuSbO_4_ setting in above ~13 T and extending to ${\mu }_{0}H\gtrsim 20\,{\rm{T}}$. Altogether, our experimental observations and model calculations provide strong arguments to identify a long-sought nematic state in LiCuSbO_4_ and stress the importance of anisotropic exchange for its relevance.

## Results

### Magnetization measurements

**Figure 2:**
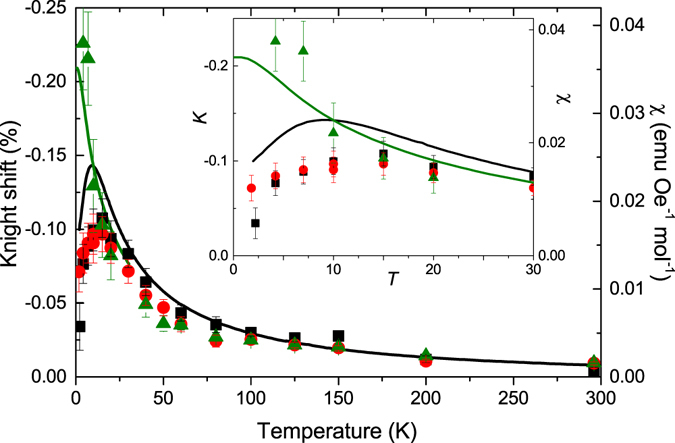
Scaling of Knight shift (*K*(3 T) ◾, *K*(9 T) , and *K*(15 T) ) and macroscopic susceptibility (*χ*(3 T) **—**, *χ*(16 T) ). The susceptibility at 16 Tesla has been reproduced from Dutton *et al*.^26^.

**Figure 3:**
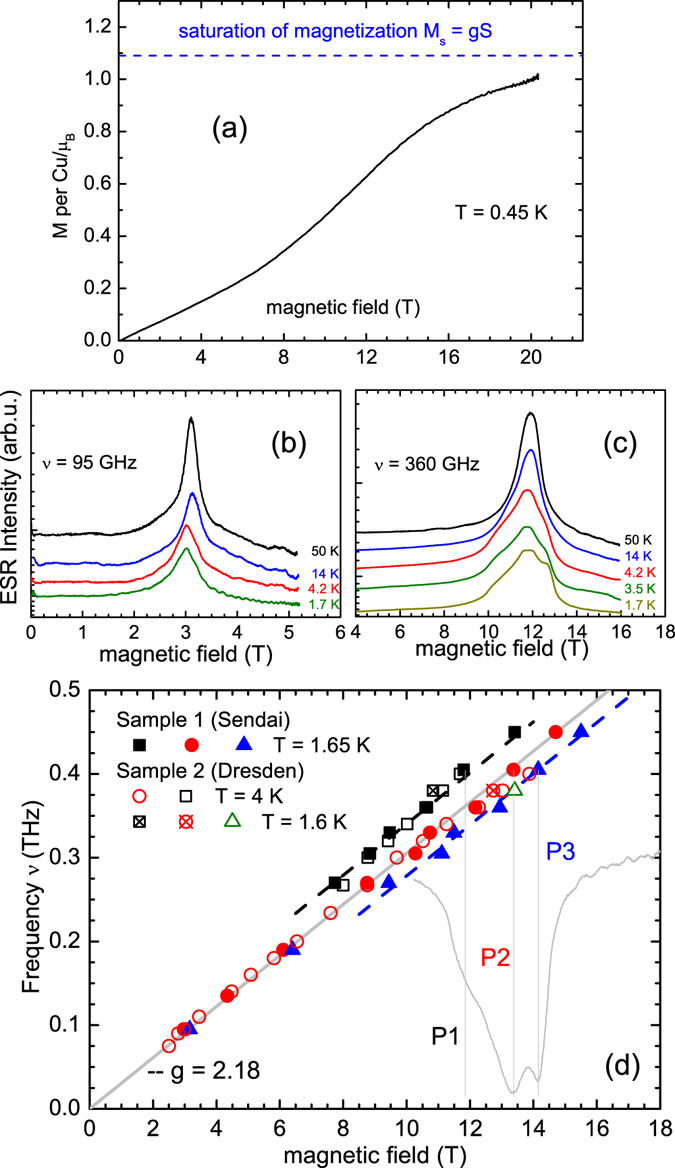
Field dependence of the magnetization at *T* = 0.45 K (**a**). Selected ESR spectra at different temperatures at frequencies *ν* = 95 GHz (**b**), and *ν* = 360 GHz (**c**), and a summary of the frequency vs. field dependence of the ESR peaks at a low *T* plotted together with a spectrum at *ν* = 405 GHz (**d**). Note the satellite peaks in the spectra in panel (c) that develop at *T* < 20 K. In (**d**), symbols - experimental peak positions P1, P2 and P3, solid and dashed lines are linear fits. The main peak P2 follows a linear gapless branch ${\nu }_{2}=({\mu }_{{\rm{B}}}/h)gH$ with *g* = 2.18, whereas the satellite branches P1 and P3 ${\nu }_{\mathrm{1,3}}={{\rm{\Delta }}}_{\mathrm{1,3}}+({\mu }_{{\rm{B}}}/h)gH$ exhibit offsets ${{\rm{\Delta }}}_{1}\approx 35\,{\rm{GHz}}$ and ${{\rm{\Delta }}}_{3}\approx -27\,{\rm{GHz}}$. (see the text).

The *T*-dependence of the static magnetic susceptibility $\chi (T)=M/H$ of LiCuSbO_4_ measured at a field *μ*_0_ *H* = 3 T is shown in Fig. 2. It appears to be in accord with the data of ref. 26. In particular, a characteristic maximum of *χ*(*T*) is observed at ~9 K. The dependence of the static magnetization *M* on the magnetic field *H* measured in pulsed fields up to 20 T at a low *T* = 0.45 K is shown in Fig. 3(a). The *M*(*H*) curve exhibits a characteristic *S*-shape. At fields below ~6 T *M* increases almost linearly and develops an upward curvature at higher fields as expected for a simple isotropic 1D-chain^29^. However, by approaching ~12 T the *M*(*H*) dependence weakens but surprisingly *no* saturation is observed up to the highest field of 20 T. Such a peculiar *M*(*H*) dependence appears to be a remarkable feature of LiCuSbO_4_ as discussed in detail below. (Linarite and some other anisotropic CuO_2_ chain systems with a low crystallographic symmetry^30^ show the similar feature but at unconvenient for experimental studies higher fields above 30 T in the latter case).

### ESR measurements

High-field ESR spectra of LiCuSbO_4_ at all measured frequencies at temperatures *T* > 20 K consist of a symmetrical single line with the shape close to a Lorentzian [Fig. 3(b,c)]. Considering the polycrystalline form of our samples, this suggests that the anisotropy of the *g*-factor, which otherwise produces a characteristic asymmetric pattern of the spectrum^31^, is smaller than the width of the signal. The *g*-factor of 2.18 obtained from the slope of the *ν*(*H*) dependence $g=(h/{\mu }_{{\rm{B}}})\,\nu /H$ [Fig. 3(d)] is a typical powder-averaged value for a Cu^2+^ ion in a distorted octahedral ligand coordination^32^. At *ν* = 95 GHz which corresponds to the resonance field ${\mu }_{0}{H}_{{\rm{res}}}\approx 3.1\,\,{\rm{T}}$ the signal remains practically a single line down to the lowest temperature. At $T\mathop{ < }\limits_{ \tilde {}}20\,{\rm{K}}$ it merely exhibits a rather small shift to smaller fields and slightly broadens, but shows no indication of an onset of a static magnetic ordering. In contrast, at *ν* ≥ 270 GHz and ${\mu }_{0}{H}_{{\rm{res}}}\ge 8.8\,\,{\rm{T}}$ the satellite peaks P1 and P3 begin to develop besides the central peak P2 at $T\mathop{ < }\limits_{ \tilde {}}20\,{\rm{K}}$ [Fig. 3(c,d)]. Their offset of ~0.8–1 T from the main peak remains practically constant, whereas the intensity increases with increasing *ν* (and *H*) and decreasing *T*. The frequency vs. field dependence of P1 and P3 approximately follows the relation ${\nu }_{1,3}={{\rm{\Delta }}}_{\mathrm{1,3}}+({\mu }_{{\rm{B}}}/h)\,gH$ with ${{\rm{\Delta }}}_{1}\approx 35\,{\rm{GHz}}$ and ${{\rm{\Delta }}}_{3}\approx -27\,{\rm{GHz}}$.

### NMR measurements

**Figure 4:**
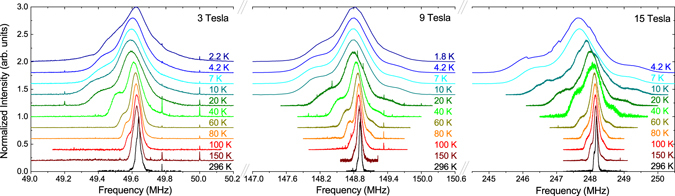
NMR spectra at 3, 9 and 15 Tesla. Note that the width of the *x*-axis is scaled by a factor 9/3 and 15/3 for 9 and 15 Tesla with respect to the *x*-axis of the 3 Tesla spectra. This way, this figure shows that the broadening is paramagnetic, and that there is no magnetic order down to ~2 K in any field.

Frequency swept ^7^Li NMR spectra in a field of 3, 9, and 15 Tesla are shown in Fig. 4 (see methods). The width of the spectra (square root of the second moment) in 3 T and at 296 K is 26.5 kHz, indicating that any quadrupolar broadening or splitting must be significantly smaller than this value. Therefore, the shape of the powder pattern is solely determined by the anisotropic dipolar hyperfine coupling of the two different Li sites in LiCuSbO_4_ times the susceptibility in the paramagnetic state. The width of the spectra scales perfectly linear with the magnetic field, indicating that the broadening of the spectra is entirely of magnetic origin. The scaling holds also at low temperatures, evidencing the absence of magnetic ordering in all fields down to ~2 K. The dipolar hyperfine coupling tensor has been obtained by lattice sum calculation and is given in the Supplement.

The Knight shift, *K*, has been extracted from the maximum of the spectra. According to the calculated dipolar hyperfine coupling tensor, both Li sites contribute to the maximum with different elements of the tensor. *K* is plotted together with the macroscopic susceptibility, in Fig. 2. The scaling of the Knight shift with *χ*, i.e., *K* = *A*_*hyp*_ · *χ*, is good down to ~15 K. Below 15 K, the *K* shows qualitatively the same *T*-dependence but the error bars of *K* become large at low temperatures due to the strong magnetic broadening. However, the similar *T*-dependences indicate that both, *K* and *χ*, are dominated by the intrinsic susceptibility and that impurity contributions are small.

**Figure 5:**
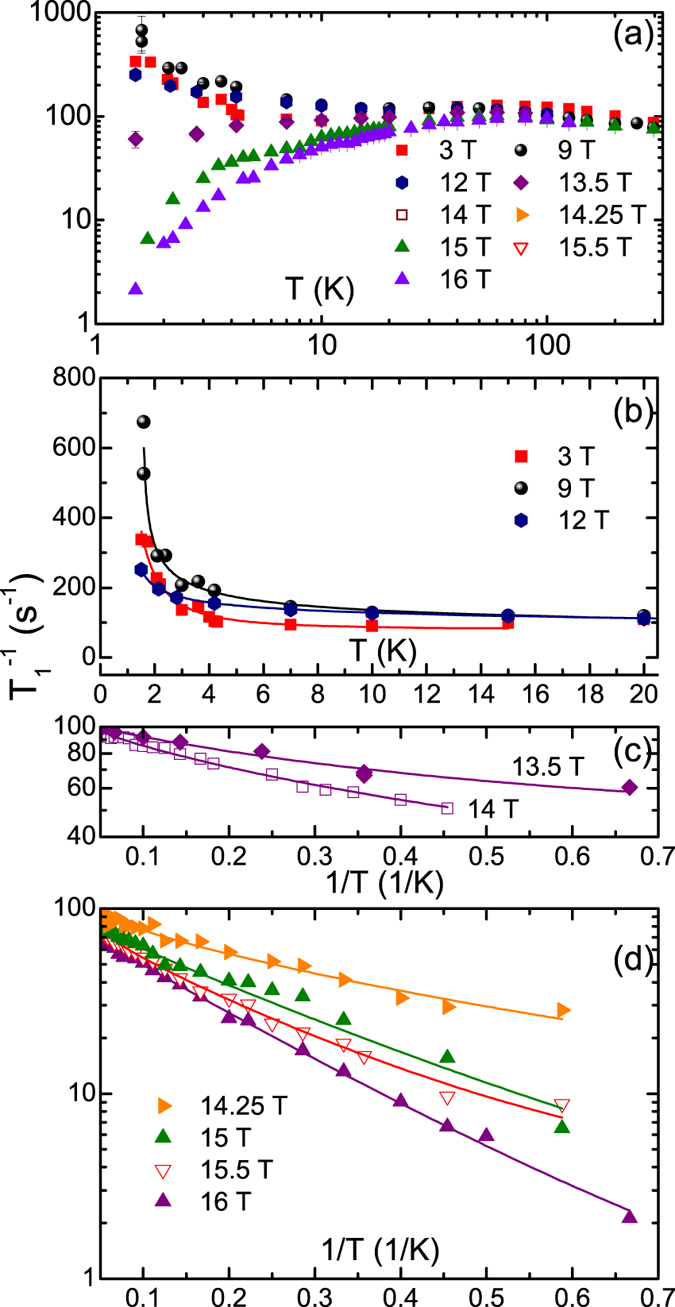
(**a**) ${T}_{1}^{-1}$ vs. temperature for different magnetic fields; (**b**) The ${T}_{1}^{-1}$ (*T*) dependence at *T* < 20 K for 3 T, 9 T and 12 T; (**c**,**d**) ${T}_{1}^{-1}$ vs. inverse temperature *T* ^−1^ at *T* < 20 K for fields >13 T. Solid lines in (**b**–**d**) are model curves according to Eq. (3).

The results of the measurements of the ^7^Li nuclear spin-lattice relaxation rate ${T}_{1}^{-1}$ at the central peak of the NMR spectrum^33^ are shown in Fig. 5. In Fig. 5(a) ${T}_{1}^{-1}$ vs. temperature is plotted on a log-log scale for six different fields from 3 to 16 Tesla. At high *T*, ${T}_{1}^{-1}$ is almost constant and field-independent as expected for a 1D spin system. However, at low *T*, below ~30 K, a dramatic field dependence develops, resulting in a difference of almost three orders of magnitude between 3 and 16 T at the lowest temperatures. For low fields below 12 T (Fig. 5(b)), ${T}_{1}^{-1}$ diverges at low temperatures (for 3 T, ${T}_{1}^{-1}$ increases below 7 K, for 9 and 12 T, ${T}_{1}^{-1}$ increases already below 15 K). In contrast, for fields above 13.5 T, ${T}_{1}^{-1}$ decreases almost exponentially at low *T*. This is highlighted in Fig. 5(c,d), where ${T}_{1}^{-1}$ is plotted vs. the inverse temperature *T* ^−1^.

## Discussion

The central experimental result of this work is an observation of distinct temperature and magnetic field dependences of the ^7^Li NMR relaxation rate ${T}_{1}^{-1}$ in the short-range ordered (SRO) state of LiCuSbO_4_ below ~10 K, as shown in Fig. 5, which will be discussed in the following.

### NMR relaxation in magnetic solids

In magnetic materials, the nuclear spin lattice relaxation rate, ${T}_{1}^{-1}$, is typically caused by the transverse (i.e. ⊥ to the nuclear spin quantization axis) components of the time-dependent fluctuating field exerted on the nuclei by the electron spin system. It can be expressed in terms of the Fourier transforms ${S}^{z,\pm }(q,\omega )$ of the time-dependent longitudinal and transverse spin-spin correlation functions $\langle {S}_{j}^{z}(t){S}_{0}^{z}\mathrm{(0)}\rangle$, $\langle {S}_{j}^{+}(t){S}_{0}^{-}\mathrm{(0)}\rangle$ and $\langle {S}_{j}^{-}(t){S}_{0}^{+}\mathrm{(0)}\rangle$, respectively^34^:

1 $$\begin{array}{rcl}{S}^{z,\pm }(q,\omega ) & = & \sum _{j}\,{e}^{-iqj}{\int }_{-\infty }^{\infty }\,dt{e}^{i\omega t}{\langle {S}_{j}^{z,\pm }(t){S}_{0}^{z,\mp }\mathrm{(0)}\rangle }_{T},\\ \quad \quad \,\,\,{T}_{1}^{-1} & \propto  & \frac{{\gamma }_{n}^{2}}{{\gamma }_{e}^{2}}\mathop{\mathrm{lim}}\limits_{\omega \to 0}\sum _{q}\,{|{A}_{\parallel }(q)|}^{2}{S}^{z}(q,\omega )\\  &  & +{|{A}_{\perp }(q)|}^{2}[{S}^{\pm }(q,\omega )+{S}^{\mp }(q,\omega )].\end{array}$$

Here *q* is the wave vector, ${\langle \cdots \rangle }_{T}$ means the thermal average, *γ*_*e*/*n*_ are the gyromagnetic ratios of the electron and the probed nucleus, and ${A}_{\parallel ,\perp }(\overrightarrow{q})$ are the hyperfine form factors of the probed nucleus. The subscripts $\parallel$ and $\perp$ denote the hyperfine tensor components generating the transversal components of the fluctuating local field at the nuclear site due to the longitudinal 〈*zz*〉 and transversal 〈+ −〉 spin-spin correlations, respectively. For $\hslash {\omega }_{{\rm{NMR}}}\ll {k}_{{\rm{B}}}T$, ${S}^{z,\pm }(q,\omega )\propto {\chi }_{z,\pm }^{^{\prime\prime} }(q,\omega ){k}_{{\rm{B}}}T/\hslash {\omega }_{{\rm{NMR}}}$, where ${\chi }_{z,\pm }^{^{\prime\prime} }(q,\omega )$ is the imaginary part of the dynamical electron spin susceptibility, ${\omega }_{{\rm{NMR}}}$ is the NMR frequency, *k*_B_ and *ħ* are the Boltzmann’s constant and the reduced Planck constant, respectively. Thus at small NMR frequencies, $1/{T}_{1}\propto {\sum }_{q}\,\chi ^{\prime\prime} (q,\omega \to 0)T$.

Generally, filtering effects may occur such that the hyperfine coupling is peaked (or zero) for certain *q*-vectors. However, the crystal structure of LiCuSbO_4_ indicates that both Li sites are coupled to several Cu sites from different chains, suggesting a rather weak dependence of ${A}_{\parallel ,\perp }$ on *q*. Since the coupling between the ^7^Li nuclear spin and the Cu spins is of dipolar nature both the longitudinal and the transversal terms in Eq. (1) are expected to contribute to the relaxation. Indeed, our estimates with the dipolar hyperfine model have revealed comparable contributions from 〈*zz*〉 and 〈+ −〉 correlations to the transversal field at both Li sites (see, Suppl.).

### Field dependence of T_1_^−1^

In weakly coupled unfrustrated critical simple AFM Heisenberg chains in the paramagnetic state far above the Neél ordering temperature ${T}_{{\rm{N}}}\ll T\ll J/{k}_{{\rm{B}}}$, the rate ${T}_{1}^{-1}$ in general *continuously increases* with decreasing *T* and/or increasing the magnetic field *H* up to the saturation field and tends to diverge by approaching *T*_N_. This is mainly due to the growth of the $\langle {S}_{j}^{+}(t){S}_{0}^{-}\mathrm{(0)}\rangle$ correlation function with increasing *H* and decreasing *T* whereas $\langle {S}_{j}^{z}(t){S}_{0}^{z}\mathrm{(0)}\rangle$ decays smoothly following a power law^35–37^.

In LiCuSbO_4_, however, ${T}_{1}^{-1}$ shows a non-monotonous and even contrasting behavior with respect to temperature and magnetic field. At relatively small fields the low-temperature region is determined by a more or less sharp increase of ${T}_{1}^{-1}$ (*T*) (Fig. 5) pointing to the vicinity of a critical magnetically ordered state at a lower temperature. Especially at ﻿﻿*μ*_*0*_ ﻿*H* = 9 T the increase is substantially more pronounced than at lower fields such as for 3 T indicating an increase of the ordering temperature of the presumable magnetic phase. Such a behavior is not expected for an ordinary antiferromagnetic Neél state where *T*_N_ is usually suppressed by an external magnetic field. In fact, the field region around 9 T is also identified by the low-temperature anomaly in the magnetic specific heat in ref. 26. It has been conjectured in that work to be a signature of an unusual field-induced magnetic phase in LiCuSbO_4_.

**Figure 6:**
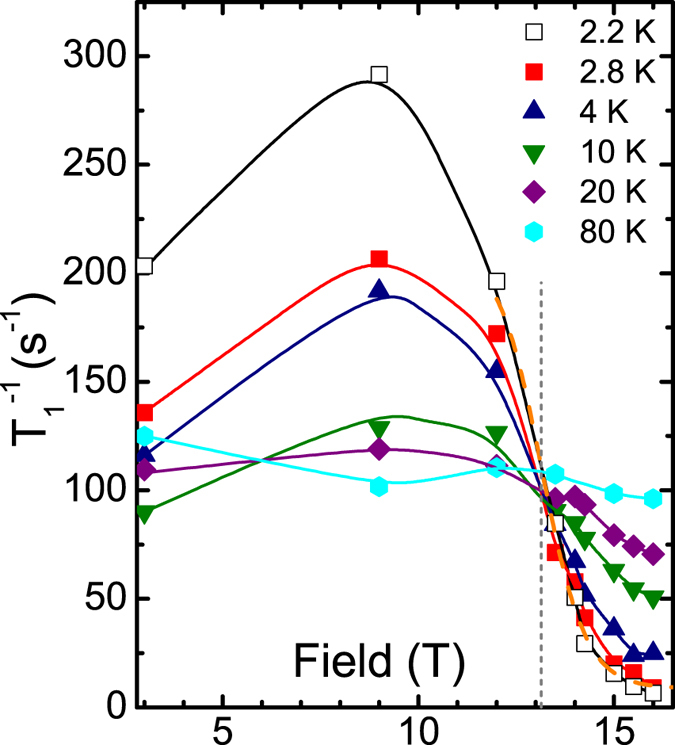
The extracted spin-lattice relaxation rate 1/*T*_1_ vs. external magnetic field for selected temperatures. Full lines are guides for the eye. The isosbestic point (IBP) near ~13 T is indicated by the vertical dashed line. The dashed orange curve is the fit to Eq. (2). (see the text).

One further and even more striking feature, which can be easily recognized in Fig. 5, is the occurrence of a threshold field of ~13 T that separates the upturn behavior from a drastic suppression of ${T}_{1}^{-1}$ vs. *T*. Indeed, plotting these data points for fixed temperatures as a function of the field, yields a set of curves with a sharp general crossing point at ${\mu }_{0}{H}_{{\rm{c}}1}\approx 13\,{\rm{T}}$, usually denoted in the literature as an *isosbestic* point (IBP)^38^ (Fig. 6). Since the nuclear ${T}_{1}^{-1}$ is governed by fluctuating fields at the nuclear site produced by electron spins, the IBP at 13 T may be identified as the critical field that separates two regimes with *different* types of magnetic fluctuations to be discussed in detail below. Indeed, at low-temperatures *T* ≤ 10–15 K which are of special interest here, the observed IBP coincides also with an inflection point (IFP). Using the generic linear field dependence $1/{T}_{1}-const\propto (H-{H}_{{\rm{c}}1})$ in the vicinity of an IFP at *H*_c1_, it is tempting to generalize that linear behavior further into the nonlinear region at higher fields employing a quasi-exponential expression that captures also the field region slightly smaller than *H*_c1_:

2 $${T}_{1}^{-1}(H)=\frac{1}{{T}_{1}\,({H}_{{\rm{c}}1})}\,[1+0.92\,\tanh \,\frac{H-{H}_{{\rm{c}}1}}{1.12A}].$$

Here $1/{T}_{1}({\mu }_{0}{H}_{{\rm{c}}1}=13\,{\rm{T}})=110\,{{\rm{s}}}^{-1}$ and *A* is a dimensional constant taken as 1 T. As can be seen in Fig. 6, Eq. (2) describes the data in the considered field region for the lowest available temperature *T* = 2.2 K quite well. This way we arrive at a smooth transition across *H*_c1_ to a pronounced exponential-type behavior at high magnetic fields and low temperature.

### Temperature dependence of T_1_^−1^

Importantly, as can be seen in a logarithmic plot of ${T}_{1}^{-1}$ vs. *T* ^−1^ in Fig. 5(c,d), a similar predominantly exponential behavior develops for the strongest fields also in the *T*-dependence of ${T}_{1}^{-1}$ suggesting the opening of an energy gap for spin excitations. For a consistent analysis of the whole set of experimental ${T}_{1}^{-1}$ (*T*) curves we have used a phenomenological combined gapped and power-law ansatz which takes into account theoretical predictions in refs 36 and 37 (see discussion below):

3 $${T}_{1}^{-1}(T)={C}_{1}(H)\,\exp \,(-{\rm{\Delta }}/T)+{C}_{2}(H)\,{(T-{T}_{c})}^{\beta }.$$

**Figure 7:**
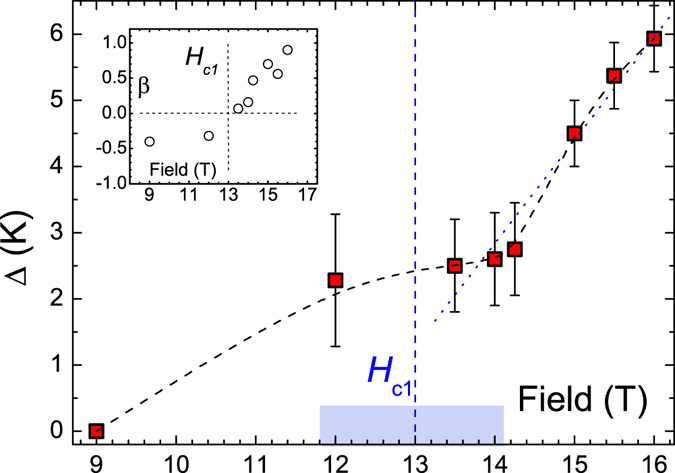
Magnetic field dependence of the gap Δ (squares) evaluated using Eq. (3). Dashed line connecting the data points is guide for the eye. Dotted line is a linear fit revealing the slope Δ/*H* = 1.56 K/T; Inset: the same for the exponent *β* in Eq. (3) (circles). Vertical dashed lines denote the isosbestic point *H*_c1_ ≈ 13 T. Shaded bar indicates a crossover region between the two distinct regimes of the ^7^Li *T*_1_ relaxation. (see the text).

Here, *C*_1_ and *C*_2_ are the weighting factors of the two contributions, Δ is the gap, and *T*_*c*_ and *β* are the critical temperature and the exponent of the power-law contribution, respectively. Possible *T*-dependences of the prefactors *C*_*i*_ remain unknown so far and have been not considered here. Though polycrystallinity of the samples and a certain distribution of the relaxation rates at highest fields and lowest temperatures (Suppl., Fig. S2) are complicating factors for the analysis, the ${T}_{1}^{-1}$ (*T*) dependences for all applied fields can be consistently modelled yielding a good description of the experimental data as shown in Fig. 5. The field dependences of the parameters of Eq. (3) are plotted in the Suppl., Fig. S3. The critical power-law behavior of ${T}_{1}^{-1}$ (*T*) at 3 T which sets in at $T\mathop{ < }\limits_{ \tilde {}}7\,{\rm{K}}$ is fully consistent with the growth of the short-ranged incommensurate correlations reported for this temperature regime in ref. 26. The fit requires a very small *T*_*c*_ ~ 0.2 K suggesting that the 3D long-range magnetic order, if present at all, is shifted to very low temperatures. At 9 T, however, *T*_*c*_ is pushed up to ~1.5 K indicating the proximity to a new, field-induced magnetic state that has been revealed in the specific heat data in ref. 26. Further increase of the field up to 12 T yields a reduction of *T*_*c*_ down to ~1 K, again consistent with the fading of the magnetic anomaly in the specific heat^26^. Interestingly, the best agreement with experiment for *H* = 12 T requires a non-zero gap value Δ ~ 2 K in the first term of Eq. (3) implying the contrasting gapped and critical power-law contributions to ${T}_{1}^{-1}$ (*T*) with Δ > 0 and *β* < 1. By crossing the IBP ${\mu }_{0}{H}_{{\rm{c}}1}\approx 13\,{\rm{T}}$ the critical growth of ${T}_{1}^{-1}$ (*T*) by lowering *T* turns into a decay corresponding to the sign change of the exponent *β* (Fig. 7, inset). Concomitantly the weight *C*_1_ of the gapped term in Eq. (3) increases on expense of the decreasing weight *C*_2_ of the power-law term (Suppl., Fig. S3). At the same time Δ increases non-linearly (Fig. 7). Despite the smallness of the weighting factor *C*_2_ at *H* > *H*_c1_, the finite power law contribution *T* ^*β*^ in Eq. (3) with positive *β*, in contrast to *β* < 0 for *H* < *H*_c1_, is required to achieve the best fit of ${T}_{1}^{-1}$ (*T*).

### Exclusion of an ordinary spin gap

**Figure 8:**
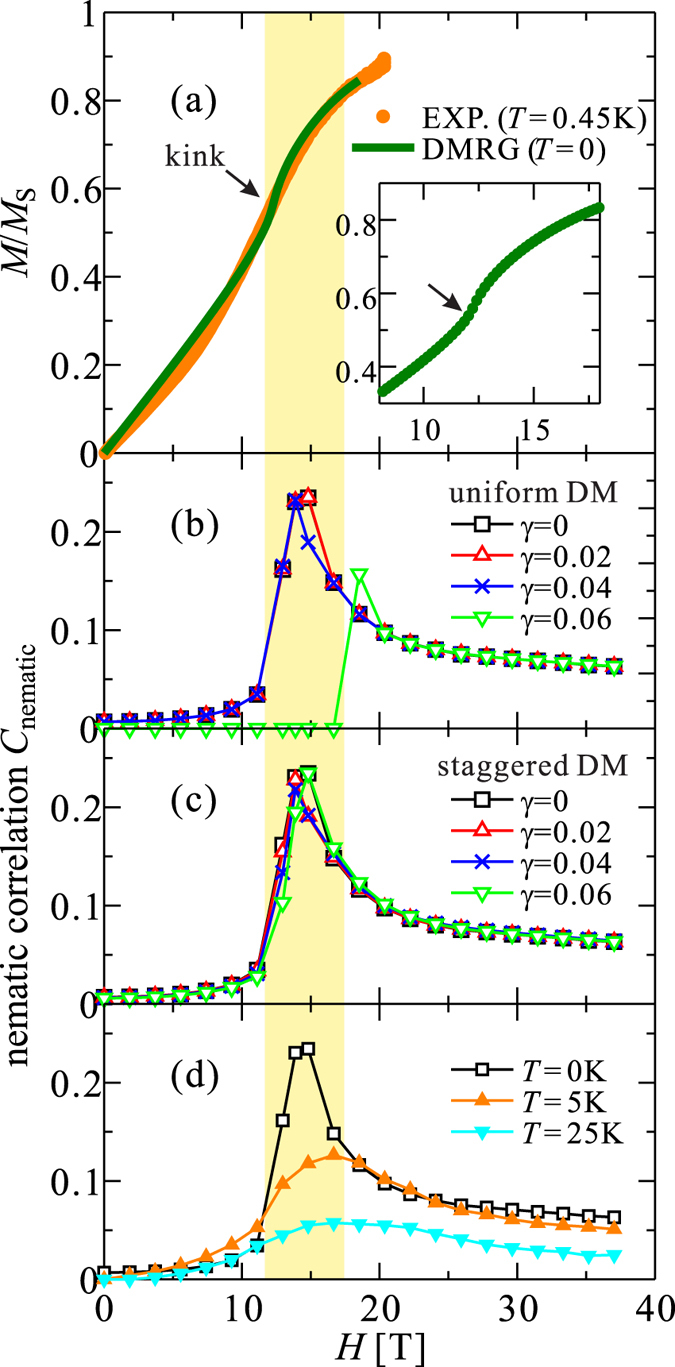
(**a**) Experimental magnetization curve at 0.45 K and theoretical magnetization curve calculated for $({J}_{1}^{x},{J}_{1}^{y},{J}_{1}^{z})=(-1.07,-0.99,-1)$ and *J*_2_ = 0.28. Inset: enlarged figure around kink in the theoretical curve. (**b**) Nematic correlation for a homogeneous DM coupling *γ* = 0, 0.02, 0.04, and 0.06. (**c**) The same for a staggered DM coupling. (**d**) Temperature dependence of the nematic correlation. The shaded area depicts the field range where a spin gap was observed in the NMR experiment.

In principle, there is a variety of conventional reasons for the opening of a gap in the spin excitation spectrum of a quantum magnet exposed to an external field. Generally, above saturation where the spins are fully polarized, all excitations acquire a gap that linearly scales with *H*. In the frustrated *J*_1_(FM) − *J*_2_(AFM) chain a two-magnon excitation is expected to have the lowest energy (see, e.g., ref. 16). In the case of LiCuSbO_4_, neglecting the non-linearity of the Δ(*H*) dependence in the crossover region, the increase of Δ amounts to Δ/μ_0_ *H* = 1.56 K/T (Fig. 7). This slope is in accord with the Zeeman energy of the flip of a single spin, i.e. a one-magnon excitation, which with the *g*-factor *g* = 2.18 obtained in the ESR experiment would amount to *gμ*_B_/*μ*_0_ *k*_B_ = 1.47 K/T. Correspondingly, the two-magnon slope should be ~3 K/T. Anyhow, we note that it would be unreasonable to identify ${\mu }_{0}{H}_{{\rm{c}}1}\approx 13\,{\rm{T}}$ as an effective saturation field. Our measurements of the static magnetization at very low *T* did not reveal a saturation of *M*(*H*) even in 20 T [Fig. 3(a)]. This finding is supported by our DMRG results (see below) showing that in a situation of the symmetric exchange anisotropy present in LiCuSbO_4_, there is no well defined saturation field at all in a literal sense, i.e. the full saturation at *T* = 0 is achieved only asymptotically [Fig. 8(a)].

Another possible reason for a field induced gap could be the presence of staggered antisymmetric Dzyaloshinskii-Moriya (DM) interactions. Due to the low crystallographic symmetry, various DM interactions are generally allowed in LiCuSbO_4_ (see below). Their magnitude in LiCuSbO_4_ can be judged from the ESR data, because ESR is very sensitive to magnetic anisotropies. Assuming that the strongest antisymmetric DM coupling is present for the intra-chain NN bond with the DM vectors perpendicular to the Cu chain (see Fig. 1 and Suppl.), a strongly anisotropic gap should open for fields applied along the chain^39^. Such a gap results in a shift of the ESR signal for this field direction proportional to *H* ^3^ at low temperatures $T < J/{k}_{{\rm{B}}}$ ^40, 41^. However, experimentally the frequency vs. magnetic field dependence of the ESR signal is linear within the error bars over a broad field range [Fig. 3(c)] which suggests that the staggered DM component of the antisymmetric exchange is small in LiCuSbO_4_. The uniform component of the DM exchange can give rise to a field independent anisotropic gap which for certain field orientations may yield a splitting of the ESR signal^42, 43^. Such a fine structure of a powder ESR spectrum of LiCuSbO_4_ is indeed found at high fields [Fig. 3(c)]. Its extend of the order ≈±30 GHz = ±1.5 K could give then the energy scale of the uniform DM component which is of a percent order of the isotropic and symmetric anisotropic exchange couplings as estimated from the magnetization data (see below).

### Evidence for spin-nematicity

Ruling out the above discussed ordinary grounds for the field-dependent spin gap in LiCuSbO_4_ enables us now to focus on a possible, more sophisticated reason for the gap opening by approaching the IBP ${\mu }_{0}{H}_{{\rm{c}}1}\approx 13\,{\rm{T}}$ from the low-field side. According to the proposed theoretical precursor phase diagram of the isotropic frustrated *J*_1_ − *J*_2_ spin chain^11, 12, 14^, sufficiently high magnetic fields yet smaller than the saturation field induce a multicomponent spin liquid including multi-magnon bound states. The two-magnon bound state (*p* = 2), corresponding to a precursor of a quadrupolar (spin-nematic) phase with a finite four-spin correlation function $\langle {S}_{j}^{+}{S}_{j+1}^{+}{S}_{0}^{-}{S}_{1}^{-}\rangle$ is the simplest one. At the lower side of this field region a collinear and incommensurate quasi long-range ordered SDW_2_-phase is stabilized since $\langle {S}_{j}^{z}{S}_{0}^{z}\rangle$ is the slowest decaying correlator. At the higher field side, above a certain crossover field, the quadrupolar 〈+ + − −〉 correlations might become nevertheless dominant yielding a competing quasi long-range ordered pronounced spin-nematic state^11, 12, 14^. In both SDW_2_ and the spin-nematic parts of the quadrupolar TL liquid, the transverse spin correlation function $\langle {S}_{j}^{+}{S}_{0}^{-}\rangle$ is expected to be gapped. This was demonstrated qualitatively for the special quasi-2D isotropic model case at *T* = 0^17^ which might probably hold in the case of relevant 3D interchain couplings at finite *T*, too. Finally, at very low fields magnon bound states as well as the collinear SDW fluctuations/order will be suppressed. Instead, a vector chiral order, which typically arises in a spin chain due to magnetic frustration somewhat modified quantitatively by possible DM couplings, turns out to be the ground state. In this phase the gap closes and the transverse $\langle {S}_{j}^{+}{S}_{0}^{-}\rangle$ correlation becomes dominant.

Since quadrupolar correlations do not generate any fluctuating fields at a nuclear site, Sato *et al*.^36, 37^ have proposed an indirect way to identify the quadrupolar phase of the isotropic *J*_1_(FM) − *J*_2_(AFM) chain and to distinguish between its SDW_2_ and the spin-nematic dominated regions. It is predicted that ${T}_{1}^{-1}$ due to longitudinal 〈*zz*〉 correlations should follow the power law ~*T* ^2*κ*−1^, where *κ* is the TL parameter. In the SDW_2_ precursor phase *κ* < 1/2 and ${T}_{1}^{-1}$ diverges with decreasing temperature whereas in the spin-nematic state *κ* > 1/2 and ${T}_{1}^{-1}$ decays as *T* → 0. In both regimes transverse 〈+ −〉 correlations yield a gapped contribution to ${T}_{1}^{-1}$ ~ exp(−Δ/*T*).

It is reasonable to attribute incommensurate spin correlations observed in LiCuSbO_4_ as well as a weak magnetic anomaly in the specific heat at low fields^26^ with the onset of a conventional short-range ordered vector chiral phase. The proximity to this phase is reflected in an increase of the ^7^Li relaxation rate at low *T* due to the growth of 〈+ −〉 correlations. From the fit with Eq. (3) a long-range vector chiral order due to interchain coupling could be realized only at very low temperatures *T*_*c*_ ~ 0.2 K and in fact was not observed down to 0.1 K^26^. A new field-induced magnetic phase at higher fields and higher temperatures yielding a strong anomaly in the magnetic specific heat^26^ can thus be naturally ascribed to the short-range ordered SDW_2_ phase. In this regime the observed strong enhancement of ${T}_{1}^{-1}$ (*T*) should be due to the dominant longitudinal 〈*zz*〉 correlations which according to the modelling of the 9 T data with Eq. (3) should yield a long-range SDW_2_ order below *T*_*c*_ ~ 1.5 K.

Further increasing the field up to 12 T yields a weakening of the 〈*zz*〉 power law contribution on which background a gapped 〈+ −〉 contribution to the ${T}_{1}^{-1}$ (*T*) dependence with Δ ~ 2 K becomes distinguishable. This clearly suggests the destabilization of the SDW_2_ state which is also reflected in the decreased value of *T*_*c*_ ~ 1 K in the model dependence (Suppl., Fig. S3).

**Figure 9:**
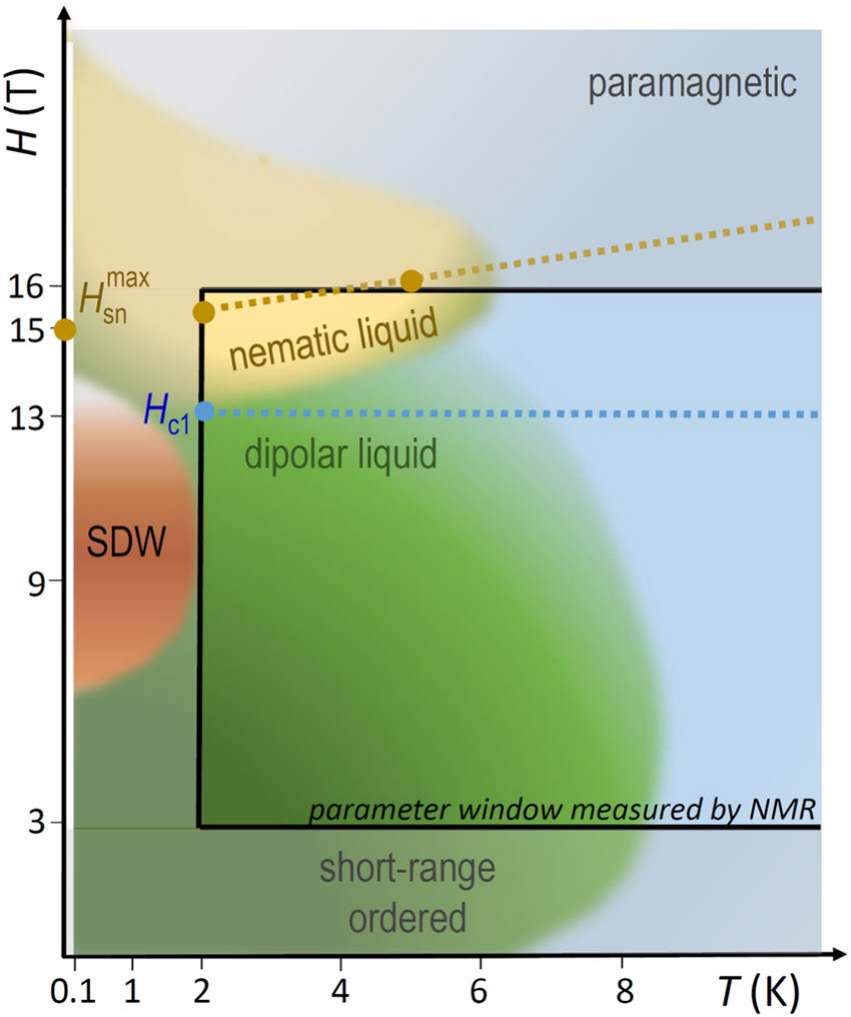
Schematic phase diagram of LiCuSbO_4_. Blue, dark green, and dark red regions reproduce approximately the diagram of Dutton *et al*. based on the analysis of the specific heat and magnetization data (Fig. 3 in ref. 26). The dark red area is suggested to present an anomalous SDW phase, whereas the dark yellow area depicts an envisaged stability region of the proposed nematic state. The region measured by the NMR in the present work is marked by the black rectangle. The blue dashed line denotes the isosbestic field *H*_c1_ (cf. Fig. 6). The brown closed circles labelled ${H}_{{\rm{sn}}}^{max}$ connected with the dashed line depict the field of the maximum of the spin-nematic correlation function as found in the DMRG analysis (cf. Fig. 8).

The vanishing of the power-law contribution at the critical IBP *H*_c1_ ≈ 13 T signals then a crossover to the distinctive spin-nematic state with dominant quadrupolar 〈+ + − −〉 correlations. In this regime the 〈*zz*〉 correlations are decaying with lowering *T* which corresponds to the sign change of the power-law exponent *κ* ^36, 37^. This is indeed the case for LiCuSbO_4_ (Fig. 7, inset). The now decaying power law contribution to ${T}_{1}^{-1}$ loses progressively its weight with increasing field whereas the gapped contribution becomes dominant (Suppl., Fig. S3). Indeed, as it has been emphasized in ref. 17 the gapped excitation spectrum is a distinct feature of the spin-nematic state of the weakly coupled 1D-chains with only a weak soft mode in the longitudinal 〈*zz*〉 channel^17, 44^. Thus, regardless of the specific details the above analysis points at a rather broad stability range of the spin-nematic liquid state in LiCuSbO_4_ above the narrow crossover region at the IBP field *μ*_0_ *H*_c1_ ≈ 13 T. This state can be considered as a precursor of the envisaged spin nematic long-range ordered phase likely to occur in LiCuSbO_4_ at comparable magnetic fields and at still lower temperatures beyond those available in the present study. The SDW_2_ and the spin-nematic states, as well as the isosbestic field *H*_c1_ and the parameter window measured by NMR are visualized in the schematic phase diagram of LiCuSbO_4_ in Fig. 9. Certainly, there must be also a second “upper” critical field *H*_c2_ framing the stability region of the strong nematic state in LiCuSbO_4_. This calls for further experimental studies of LiCuSbO_4_ at higher fields and also at lower temperatures beyond the scope of the present work.

### Band structure calculations

As in other related materials (see., e.g., refs 20–22 and 27), the edge-sharing geometry of the CuO_4_ plaquettes in the CuO_2_ chains in LiCuSbO_4_ (Fig. 1) is expected to give rise to the usual frustrated magnetism due to the presence of oxygen mediated frustrated AFM NNN intra-chain couplings. The nearly 90° Cu-O-Cu bond angle, i.e. 93°, points to FM NN intra-chain interactions due to the presence of a sizable direct FM coupling *K*_*pd*_ ~ 100 meV between two holes residing on neighboring sites with Cu 3*d* and O 2*p* orbitals and a significant compensation of the AFM NN superexchange contributions.

We have performed DFT and DFT+*U* band structure calculations with the aim to understand (i) the amount of interchain couplings and (ii) the magnitude of the intra-chain couplings. With respect to (i) we have analyzed the dispersion of bands and found pronounced 1D van Hove singularities near the Fermi level. Thus, we have confirmed the nearly 1D behavior of LiCuSbO_4_. Then, in general, the exchange coupling strength can be estimated simply by the AFM contribution ${J}_{1}=4{t}_{1}^{2}/{U}_{{\rm{eff}}}$, where ${U}_{{\rm{eff}}}\sim {{\rm{\Delta }}}_{pd}\approx 3$ to 4 eV is the effective Coulomb repulsion within a *single*-band type approach for the Cu-sites with NN transfer integral *t*_1_. The frustrating *J*_2_ is measured by the analogous expression using the NNN transfer integral *t*_2_ instead ignoring a much weaker direct FM contribution as compared to that of the NN bond (${K}_{pd}\gg {K}_{pp}$) and the small hole occupation at O 2*p* orbitals. Note that applying such a simple model to charge transfer insulators as cuprates, one is left with an effective onsite repulsion *U* of the order of the Cu 3*d*-O 2*p* onsite energy difference which is significantly smaller than the *U*_*d*_ ~ 5.5 eV at Cu sites employed in the DFT+*U* calculations or in more sophisticated five-band *pd* Hubbard models^45, 46^ to be considered for to the case of LiCuSbO_4_ elsewhere. To check this simple first approach, we have determined the Cu-Wannier functions which contain also the essential O 2*p* contributions. Their tails point to the important coupling directions. In fact, a closer inspection of the crystal structure reveals nonequivalent “left” and “right” NN intra-chain bonds (Fig. 1). This gives rise to alternating NN transfer integrals (${t}_{1}\ne {t}_{1}^{^{\prime} }$). The one-band fit results in the following transfer integrals (given in meV): ${t}_{1}=-95.51,\,{t}_{1}^{^{\prime} }=\mathrm{56.44,}\,{t}_{2}=56.96$, and ${t}_{3}=-11.88,\,{t}_{3}^{^{\prime} }=-16.57$. Then the mean NN transfer integral ${\bar{t}}_{1}$ would provide an AFM superexchange contribution to ${J}_{1}^{e}$ for an equidistant chain of about 67 K which for a typical ${J}_{1}^{e}$ of about −80 K like in linarite (see, refs 22 and 47 and references therein) corresponds to an FM contribution of −147 K. As a result we arrive at a sizable splitting of the two NN exchange integrals: ${J}_{1}\approx -160\,{\rm{K}}$ and ${J}_{1}^{^{\prime} }\approx -90\,{\rm{K}}$, whereas *J*_2_ ≈ 37.6 K, only. Thus, within a 1D picture we are left with a dominant FM total NN coupling and an unrenormalized mean frustration parameter $\bar{\alpha }={J}_{2}/[({J}_{1}+{J}_{1}^{^{\prime} })/2]\sim 0.3$. This value is close to that (*α* = 0.28) estimated from the fitting of the observed magnetization curve by the DMRG calculations (see below).

### Symmetry analysis and the role of DM interactions

The crystal structure of LiCuSbO_4_ is described by the polar space-group Cmc2_1_^26^. The low symmetry implies modifications of the standard *J*_1_ − *J*_2_ spin-model to describe the chains of the edge-shared CuO_4_ square-like plaquettes runnning in *a*-direction. More details of our symmetry analysis are given in the Supplement. In particular, because of the low symmetry, antisymmetric Dzyaloshinskii-Moriya (DM) interactions are allowed for the NN bonds along the spin-chains, ${E}_{D}={{\bf{D}}}_{\nu }\cdot ({{\bf{S}}}_{i}\times {{\bf{S}}}_{i+\nu })$ and have *both* a homogeneous and a staggered component. These microscopic antisymmetric exchange interactions are caused by the relativistic spin-orbit interactions and compete with the isotropic exchange interactions.

To fit the experimental data for LiCuSbO_4_, Dutton *et al*.^26^ assumed in their model the presence of this exchange anisotropy for the NN bond, while neglected the anisotropic terms *linear* in |**D**_*ν*_|. However, this could yield an unrealistic picture for the basic magnetic couplings in LiCuSbO_4_. The determined strength of the effective anisotropic coupling constant is large and would imply an unusual order of magnitude $|{{\bf{D}}}_{1}|/|{J}_{1}|\sim 1$. The presence of the relativistic antisymmetric exchange in crystals belonging to the crystal classes 2 mm or *C*_2*ν*_ may give rise to two rather different states^48^: weak ferromagnetism or more generally canting of spins with a net magnetization can be derived by the staggered DM couplings. Besides, the acentric crystal structure also allows for the presence of ‘*inhomogeneous DM couplings*’^48^, that derive from the homogeneous part of the DM interaction. It is known that this type of couplings can suppress any long-range ordered states and may be related to the absence of magnetic ordering down to ~0.1 K in LiCuSbO_4_.

### DMRG-calculations

The main aim of this part is to present an analysis of a novel anisotropy mechanism based on the low-symmetric NN exchange anisotropy, which in addition to the *J*_1_ − *J*_2_ frustration, stabilizes a nematic phase in a moderate high-field region to be specified below. We present a first brief analysis also of the effect of weak homogeneous and staggered NN DM couplings which were found not to destroy the nematicity although some weakening has been observed.

Figure 8(a) shows the magnetization curve *M*(*H*) measured at *T* = 0.45 K. Noteworthy, a full saturation is not reached even in the highest accessible field of 20 T. Although this is reminiscent of a typical feature of *M*(*H*) at high temperature, the observation temperature is now low enough to prevent a significant finite-temperature effect. To provide a reasonable explanation for this feature, we introduce a 1D frustrated Heisenberg model with an *xyz* exchange anisotropy and a magnetic field *H* along the *z* axis. The Hamiltonian is then given by

4 $$\begin{array}{rcl} {\mathcal H}  & = & \sum _{i,\gamma =x,y,z}\,{J}_{1}^{\gamma }{S}_{i}^{\gamma }{S}_{i+1}^{\gamma }+{J}_{2}\sum _{i}\,{{\bf{S}}}_{i}\cdot {{\bf{S}}}_{i+2}\\  &  & +H\sum _{i}\,{S}_{i}^{z},\end{array}$$

where ${J}_{1}^{\gamma }$ and *J*_2_ are the NN FM and the NNN AFM exchange couplings, respectively, and ${S}_{i}^{\gamma }$ is the *γ*-component of the spin-operator **S**_*i*_. When $H\gg {J}_{1}^{\gamma }$, *J*_2_, by taking the fully polarized FM state as non-perturbative state, its energy is lowered by ${\rm{\Delta }}E={({J}_{1}^{x}-{J}_{1}^{y})}^{2}/\mathrm{[32(}H-{J}_{1}^{z}-{J}_{2})]$ through the second-order process of an individual double spin-flip. Therefore, the magnetization behaves like ${M}_{S}-M\propto {({J}_{1}^{x}-{J}_{1}^{y})}^{2}/H$ at high fields and saturates at its maximum value *M*_S_ only at $H=\infty$, only. We have calculated the magnetization curve using the DMRG technique. By fitting the experimental curve, we have found a possible parameter set: ${J}_{1}^{z}=-546\,{\rm{K}}$, $({J}_{1}^{x}/{J}_{1}^{z},{J}_{1}^{y}/{J}_{1}^{z})=(1.07,0.99)$, and *J*_2_ = 153 K. Note that these numbers are effective values in the 1D limit, which can be significantly different from the bare values of NN and NNN exchange couplings determined by the DFT+*U* calculations. Other contributions such as the interchain and longer-range exchange couplings are renormalized into them. As a related similar example we refer the reader to linarite^22, 47^.

More interestingly, an exotic nematic state is established by the *xyz* exchange anisotropy. The *xy* components of the first term of Eq. (4) can be divided into an exchange term $\frac{{J}_{1}^{x}+{J}_{1}^{y}}{4}({S}_{i}^{+}{S}_{i+1}^{-}+{h}.{c}\mathrm{.)}$ and a double spin-flip term $\frac{{J}_{1}^{x}-{J}_{1}^{y}}{4}({S}_{i}^{+}{S}_{i+1}^{+}+h.c\mathrm{.)}$. The latter seems to create an attractive interaction among the parallel spins. Therefore, a 2-magnon bound state, i.e., a nematic state, may be naively expected at high fields in the presence of *xyz* exchange anisotropy. To check this possibility, we have calculated the nematic correlation function as an indicator of magnon pairing

5 $$\begin{array}{rcl}{{\mathscr{C}}}_{{\rm{nematic}}} & = & \langle {S}_{i}^{-}{S}_{i+1}^{-}\rangle -\langle {S}_{i}^{-}{S}_{i+\infty }^{-}\rangle \\  & \equiv  & \langle {S}_{i}^{+}{S}_{i+1}^{+}\rangle -\langle {S}_{i}^{+}{S}_{i+\infty }^{+}\rangle .\end{array}$$

Note that this correlation vanishes for lacking *xyz* exchange anisotropy, because there is no overlap between different *S* ^*z*^ sectors. Furthermore, there is an important difference of the two-magnon instability within our unconventional nematicity scenario as compared to the usual isotropic counterpart mentioned above: namely, the total momentum *q* of a bound two-magnon pair equals to zero just as in a standard BCS superconductor whereas in the isotropic counterpart it equals to *q*_*a*_ = *π* which resembles the behavior of a Pauli-limited strongly paramagnetic superconductor in an extremely inhomogeneous Fulde-Ferrel-Larkin-Ovchinnikov (FFLO) state. In this context, the recently proposed isotropic multipolar field theory based scenario by Balents and Starykh^49^ with a two-magnon pair with a finite but very small total momentum of $0 < {q}_{a}\ll \pi$ is noteworthy. The present single chain Hamiltonian with the involved specific exchange anisotropy describes a 1D system with a distinctive nematically *ordered* ground state at *T* = 0 and at high enough magnetic fields in contrast with simple AFM Heisenberg chains. With increasing finite *T* this distinct order is more and more suppressed. The stability of the former generalized also to 2D or 3D with respect to interchain couplings and various DM couplings will be investigated in detail in forthcoming work.

The nematic correlation for our parameter set is plotted as a function of *H* in Fig. 8(b). It is significantly enhanced just above the kink position of the theoretical magnetization curve. This field range with the enhanced correlation agrees well with that region where the spin gap has been experimentally observed, namely, in between *μ*_0_ *H* = 13–16 T. A similar nematicity scenario has been proposed in our recent work devoted to linarite^22^, but there yet not fully confirmed experimentally due to a phase separation and other experimental and physical difficulties. Furthermore, we have also studied the effect of additional uniform or staggered DM couplings allowed by the crystallographic symmetry as mentioned above ${ {\mathcal H} }_{{\rm{DM}}}={\sum }_{i}\,{\bf{D}}\cdot ({{\bf{S}}}_{i}\times {{\bf{S}}}_{i+1})$ with **D** = (0, 0, *γ*). As a result we found that the nematic state is *hardly* affected by a weak DM coupling for *γ* < 0.05. The effect of a staggered DM interaction is even weaker than that of a uniform one. For simplicity, the dimerization of the NN exchange couplings, suggested by the DFT, was not taken into account in the present DMRG calculations. However, the stability of the nematic state is mostly related to the magnitude of the exchange anisotropy and is less affected by the dimerization. Also, the *T*-dependence of the correlation is plotted in Fig. 8(d). We can see that the sharp enhancement of the correlation around *μ*_0_ *H* = 13 T at *T* = 0 disappears for higher *T* which points to existence of the mentioned above upper critical field.

Thus, we are confronted with a somewhat unusual situation: the pronounced spin gap and the strongly enhanced nematic correlations are, in a literal sense, not the result of the occurrence of a novel order parameter associated with symmetry breaking due to a second order phase transition from a high temperature and low-field para-phase, since at low fields the nematic order at *T* = 0 already exists albeit at a low level. Instead, based on our calculations and in accord with the experimental data, we suggest a crossover from a weak nematic state in a narrow field range at about 13 T to a pronounced nematic state up to at least 16 T to 20 T to be followed by a broad field range where it decreases again (Figs 8 and 9).

Such transitions without a symmetry change of the macroscopic order parameter are reminiscent of mesoscopic liquid-liquid transitions in ordinary liquids such as, for instance, in phosphorus and water. It has been found that those single-component liquids may have different liquid states with distinct correlation functions. A transition between them driven by some external control parameter, such as temperature or pressure, is characterized by the *quantitative* change of a correlation function, only^50–52^. In the case of LiCuSbO_4_, with increasing *T* these changes are smeared out and the consequences of the suppressed nematic order parameter are difficult to be observed. In such a complex situation further experimental and theoretical studies beyond the scope of the present work are necessary to refine the parameters of our proposed model and to take into account explicitly the weaker couplings and modifications suggested by the real structure including also impurities or defects.

## Summary

In conclusion, we have presented strong experimental and theoretical indications of the occurrence of a distinctive spin-nematic state in the frustrated anisotropic spin chain cuprate LiCuSbO_4_. This state emerges above an isosbestic point *μ*_0_ *H*_c1_ ≈ 13 T detected in the field dependence of the ^7^Li NMR relaxation rate ${T}_{1}^{-1}$ at low temperatures. The analysis of the temperature dependences of ${T}_{1}^{-1}$ reveals that *H*_c1_ separates a lower-field regime with strong enhancement of ${T}_{1}^{-1}$ at low *T* from a higher-field regime with a sharp decrease of ${T}_{1}^{-1}$ (*T*). The former is ascribed to diverging longitudinal spin correlations typical for a multipolar SDW liquid whereas the latter is due to the power-law like decaying longitudinal and gapped transverse spin correlations characteristic of the spin-nematic liquid. Theoretical analysis justifies the occurrence of this “hidden” spin-nematic state in LiCuSbO_4_ in an extended field range above *H*_c1_. A broad range of stability of this spin-nematic state unexpected in the corresponding isotropic spin-chain model can be ascribed to the presence of exchange anisotropies. Indeed, as is found in the DMRG calculations it can be due to a special low-symmetry symmetric exchange anisotropy which is reflected in the high-field magnetization data. The missing SDW-type magnetic ordering at lower fields might be ascribed to some structural disorder and/or frustrated interchain interactions caused by DM couplings allowed in this low-symmetry crystal structure in general. The small but finite DM coupling favoring a noncollinear spin arrangement could be responsible for the suppression of the otherwise strongly competing anomalous collinear SDW_2_ phase. On the other hand, according to complete diagonalization and DMRG studies in high magnetic fields the presence of a weak DM interaction, uniform or staggered, identified in the symmetry analysis of LiCuSbO_4_ and assessed with ESR, are not detrimental for nematicity. Merely an alteration of the two NN exchange couplings may somewhat reduce the binding energy of two-magnon bound states as compared to equal NN bonds. The remarkable interplay of symmetric and antisymmetric exchange anisotropies with sizable frustration is of general interest in modern quantum magnetism and calls for deeper theoretical and experimental studies.

## Methods

### Sample synthesis and characterization

**Figure 10:**
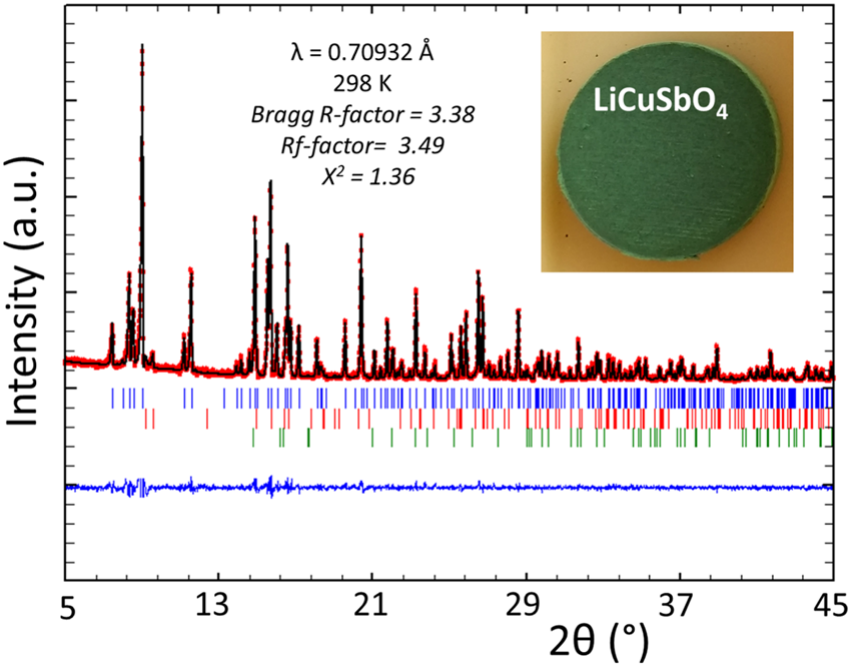
Calculated and observed X-ray diffraction pattern of the Rietveld refinement for LiCuSbO4. The difference curve is shown in blue; reflection positions are indicated by the vertical lines for LiCuSbO4 (blue), LiSbO_3_ [2.36(5) wt%] (red) and CuO [0.72(1) wt%] (green) impurity phases. ($\lambda =0.70932\,{\AA }$, Bragg R-factor: 3.38%; Rf-factor = 3.49%; Bragg R-factor = $\sum |{I}_{{\rm{ko}}}-{I}_{{\rm{kc}}}|/\sum {I}_{{\rm{ko}}}$; Rf-factor = ${[(N-P)/\sum {w}_{{\rm{i}}}{y}_{{\rm{io}}}2]}^{\mathrm{1/2}}$). The inset shows optical image of a typical green pellet of polycrystalline sample of LiCuSbO_4_ after annealing in dried oxygen flow.

Green polycrystalline sample of LiCuSbO_4_ was prepared through solid-state reaction using stoichiometric mixture of dried Li_2_CO_3_ (99.98%, Chempur), CuO (99.95%, Aldrich) and Sb_2_O_5_ (99.9%, Alfa Aesar). The mixture of the precursor compounds was homogenized by grinding in a mortar and pestle, followed by a 16 h sintering at 700 °C. The sample was subsequently grounded, pressed into pellets and fired at 1000 °C for 48 h. Pure LiCuSbO_4_ phase was obtained after annealing of the pellets at 1050 °C for 24 h under dried oxygen flow. Phase purity of the products was assessed by powder X-ray diffraction, by using a STOE Stadi P powder diffractometer with Mo K_*α*1_ radiation. The diffractometer is equipped with a curved Ge (111) monochromator and a 6° linear position sensitive detector (DECTRIS MYTHEN 1 K detector). Powder x-ray diffraction data were analyzed with the Rietveld method using the FULLPROF in the WinPlotR program package program^53^. The background was fitted using linear interpolation between selected points. The March-Dollase model for preferred orientation was used in all of the refinements, and a pseudo-Voigt function was used as the peak-shape model. As refinable parameters background, scale factor, half width, Caglioti variables (U, V, W), lattice parameters, asymmetries and the overall temperature factor were allowed. Based on Rietveld analysis of the powder x-ray diffraction data (Fig. 10), the main phase is LiCuSbO_4_ 96.93(6) wt% (orthorhombic, Cmc21, $a=\mathrm{5.7493(1)}\,\AA$, $b=\mathrm{10.8828(2)}\,\AA$, $c=\mathrm{9.7429(1)}\,\AA$). LiSbO_3_ 2.36(5) wt% (orthorhombic, Pnma, $a=\mathrm{5.1756(4)}\,\AA$, $b=\mathrm{4.9092(3)}\,\AA$, $c=\mathrm{8.4887(6)}\,\AA$) and 0.72(1) wt% CuO are two minor impurity phases. The amount of foreign phase is very similar to the previous report in ref. 26.

### Nuclear magnetic resonance

NMR spectra were obtained with a Tecmag Apollo spectrometer and a standard sample probe from NMR Service GmbH. The magnetic field has been applied by a 16 T Oxford Instruments superconducting magnet. Temperatures were regulated by a ^4^He variable temperature insert (VTI). Temperatures below 4.2 K were achieved by pumping on the VTI. At high temperatures and small fields, Fourier transformations (FFT) of the spin echo covered the whole spectral width. At lower temperatures, we swept the frequency and summed up the FFT’s to obtain the complete spectrum. The spectra at 15 T below 10 K have been obtained by field sweeps, and converting into frequency sweeps. This is easily possible due to the negligible quadrupole interaction. We have confirmed the correctness of this procedure at higher temperatures. The spin lattice relaxation rate, ${T}_{1}^{-1}$, has been measured by standard saturation recovery at the peak of the spectra. The nuclear magnetization, *M*_0_, has been saturated by a train of radio frequency pulses, before measuring the recovered nuclear magnetization, *M*(*τ*), depending on the time *τ* between the saturation train and the spin echo sequence.

### Electron spin resonance

ESR spectra were measured with the Terahertz ESR Apparatus (TESRA-IMR) installed in the magnetism division of Institute of Materials Research, Tohoku University^54^. As sources of the microwave radiation up to 450 GHz conventional Gunn oscillators were employed. The signals were detected with an InSb detector. Pulse magnetic fields up to 20 T were generated with a solenoid magnet and a 90 kJ capacitor bank. The sample temperature was regulated with a ^3^He cryostat. Additional ESR measurements were performed at the IFW Dresden with a home-made multi-frequency high-field ESR spectrometer at magnetic fields up to 16 T and at frequencies *ν* up to 400 GHz^55^. For the generation and detection of the microwave radiation millimeter wave backward oscillators and an InSb bolometer from QMC Insruments Ltd., as well as a millimeter wave network analyzer from AB Millimetre, have been used. DC magnetic fields were obtained with a solenoid superconducting magnet from Oxford Instruments equipped with a ^4^He variable temperature insert.

### Static magnetization

Temperature dependence of the static magnetic susceptibility in fields up to 5 T in the temperature range *T* = 2–300 K was measured with the SQUID magnetometer from Quantum Design. Static magnetization in fields up to 20 T was measured with a standard inductive method using compensated pickup coils and a nondestructive pulse magnet (for details see ref. 56). The sample was immersed into liquid ^3^He to reach a temperature as low as 0.45 K.

### Density functional calculations

Relativistic density functional (DFT) electronic structure calculations were performed using the full-potential local orbital FPLO code^57, 58^ (http://www.fplo.de), version fplo14.00-49. For the exchange-correlation potential, within the local density (LDA) and the the general gradient approximation (GGA) the parametrizations of Perdew-Wang^59^ and Perdew-Burke-Ernzerhof^60^ were chosen, respectively. Both exchange-correlation potential yielded essentially the same band structure. To obtain precise band structure information, the final calculations were carried out on a well converged mesh of 4800 *k*-points (20 × 20 × 12 mesh, 1332 points in the irreducible wedge of the Brillouin zone). For our calculations, we used the experimental crystal structure of ref. 26. However, to model the Li split positions Li1a (4a), Li1b (4a) and Li2 (8b), we used averaged coordinates: Li1 (4a) 0.0 0.3610 0.6974 or the coordinates of the nearby high symmetry position Li2 (4a) 0. 0.0002 0.2660; Li split positions have been successfully modeled this way for the related edge-sharing chain compound LiZrCuO_4_^61^.

## Electronic supplementary material

Supplementary information
