# Supplementary material for: Signatures of a magnetic field-induced unconventional nematic liquid in the frustrated and anisotropic spin-chain cuprate LiCuSbO4
Source: Sci Rep. 2017 Jul 27;7:6720. doi: 10.1038/s41598-017-06525-0 (PMC5532373; doi:10.1038/s41598-017-06525-0)
Supplement: Supplementary file 1 — Supplementary information [file 41598_2017_6525_MOESM1_ESM.pdf]

Signatures of a magnetic field-induced unconventional nematic liquid in the frustrated and anisotropic spin-chain cuprate $LiCuSbO_4$

H.-J. Grafe,[1] S. Nishimoto,[1, 2] M. Iakovleva,[1, 3] E. Vavilova,[1, 3] L. Spillecke,[1, 4] A. Alfonsov,[1] M.-I. Sturza,[1] S. Wurmehl,[1] H. Nojiri,[5] H. Rosner,[6] J. Richter,[7] U.K. Rößler,[1] S.-L. Drechsler,[1] V. Kataev,[1, *] and B. Büchner[1, 4]

[1]*Leibniz Institute for Solid State and Materials Research IFW-Dresden, D-01171 Dresden, Germany*
[2]*Institute for Theoretical Physics, Technical University Dresden, D-01069 Dresden, Germany*
[3]*Zavoisky Physical-Technical Institute of the Russian Academy of Sciences, 420029 Kazan, Russia*
[4]*Institute for Solid State Physics, Technical University Dresden, D-01069 Dresden, Germany*
[5]*Institute of Materials Research, Tohoku University, 980-8577, Sendai, Japan*
[6]*Max-Planck-Institute for Chemical Physics of Solids, Dresden, Germany*
[7]*Universität Magdeburg, Institut für Theoretische Physik, Germany*
(Dated: July 17, 2017)

Supplement

Details of the exchange and kinematic interactions as derived from the band structure calculations. The two tiny third neighbor interactions $J_3 \sim 1.6$ to 3.2 K, respectively, were both ignored in the DMRG calculations for the sake of simplicity, i.e. first of all to restrict the number of model parameters. Note that the NN exchange integrals may have a margin of error about a few ten K due to several uncertainties like in $U_{\rm eff}$ and a more accurate estimation is left for future work. However, the alternation of the NN exchange integrals is most likely, a rare situation so far only met for the celebrated spin-Peierls compound $CuGeO_3$ [1] with two AFM NN couplings and probably with two ferromagnetic ones for $Rb_2Cu_2Mo_3O_{12}$ [2, 3], only. The interchain transfer integrals were derived employing the calculated Wannier functions to reproduce the full DFT band dispersion of the band complex near the Fermi energy. Their overlapping tails are smaller by an order of magnitude ~ 10 meV as compared to the NN inchain values giving this way rise to still smaller frustrated AFM contributions of the order of 1 K, only, (Fig. 1 in the main text). Since there are no reasons to expect significant FM contributions, the resulting total interchain couplings are expected to be extremely small, too. This qualitative estimate might explain the strongly supppressed SDW and spiral phases and most importantly will only weakly suppress the nematic state [4].

Detailed symmetry analysis: Non-centrosymmetric crystal structure from the class C_{2v}: generalities. The crystal structure of $LiCuSbO_4$ has been described in the acentric (polar) space-group Cmc2_1 (No 36, point symmetry C_{2v}) [5]. The low symmetry allows that DM interactions,

$$E_D = \mathbf{D}_{ij} \cdot (\mathbf{S}_i \times \mathbf{S}_j), \tag{S1}$$

can occur for *all* bonds between two Cu-sites. A long-range-ordered magnetic state (or the hypothetical mean-field magnetic order) in these materials may appear as a basically AFM spin-pattern that is twisted into long-period textures. A 1D texture is known as a "Dzyaloshinskii spiral". A monodomain-state of such a spiral has a spatially fixed rotation axis w.r.t. to its propagation direction and a fixed (chiral) sense of rotation. In the crystal classes $C_{nv}, n = 2, 3, 4, 6$ the possible propagation directions $\mathbf{p}$ are perpendicular to the unique axis of the crystals $\mathbf{c}$. The propagation direction in the ground-state is determined by weaker anisotropies of the magnetic system. The rotation axis for the spins is transverse to this propagation axis and also is in the plane perpendicular to the crystallographic axis. Thus, the staggered vector rotates in a cycloidal manner within the plane spanned by the propagation direction $\mathbf{p}$ and the axis c. This slow rotation of the primary order-parameter components is accompanied by a (weak-FM) spin-density wave, similar to magnets from crystal classes ($C_{nv}, n = 3, 4, 6$) analysed in Ref. 6.

In general, Lifshitz invariants in several spatial directions do *frustrate* long-range ordering (LRO) as these terms act like a frozen gauge potential background on the incipient ordering and enhances the impact of fluctuations to destroy LRO as has been described for a wide range of different systems from cholesteric liquid crystals [7] to chiral magnets [8]. In a paramagnetic but spin-liquid state of an AFM system with C_{2v} symmetry, a possible kind of correlated low-energy excitations would consist of skyrmionic modes. These are localized excitations (possibly in the shape of ellipsoidal staggered spin-states) that are twisted in the two basal plane directions. Other possible excitation modes would be 1D kink-like solitonic units with an envelope function of the order-parameter that restricts their spatial extension on a paramagnetic background.

---

* Corresponding author: v.kataev@ifw-dresden.de

In both kinds of such non-linear excitations, the magnitude and direction of the AFM correlations within such a fluctuating excitation are intertwined and cannot be separated [8–11]. For a fully established 3D spin-order, the Lifshitz-type invariants for the vector components of a magnetic ordering mode have spatial gradients in the base-plane (perpendicular to the n-fold crystal axis) in the continuum theory for crystals from the classes C_{nv}. The very existence of strong enough frustrating DMIs will tend to suppress classical 3D long-range ordering and exacerbate the impact of quantum-fluctuations in a low-dimensional spin-system.

Experimental details NMR

Experimental determination of the hyperfine coupling: Fig. S1 shows the Knight shift K determined from the peak of the spectra in 3 T and 15 T versus the macroscopic susceptibility χ of the powder sample in 3 T. Linear fits give hyperfine coupling constants of $A_{hyp} \approx -0.27\,\mathrm{kOe}/\mu_B$, and an orbital shift of $K_{orb} = -0.005\,\%$ for 3 T and $A_{hyp} \approx -0.24\,\mathrm{kOe}/\mu_B$, and $K_{orb} = -0.002\,\%$ for 15 T. This is comparable to the value extracted in $LiCuVO_4$ for a field perpendicular to the chain direction, $A_{perp} = -0.19\,\mathrm{kOe}/\mu_B$ [12]. Anisotropic hyperfine coupling as can be determined by measurements on single crystals (see, e.g., [13, 14]) cannot be extracted since only powder susceptibility data are available.

Calculation of the hyperfine tensor: The local field of the Cu electron spins $\mathbf{S}_j$ at the nuclear spin $\mathbf{I}_i$ of the Li $\mathbf{h}_i = \sum_j \hat{A}_{ij}\langle\mathbf{S}_j\rangle$ is transferred by the dipolar hyperfine coupling tensor

$$\hat{A}_i = \sum_j \hat{A}_{ij} = \begin{pmatrix} A_i^{aa} & A_i^{ab} & A_i^{ac} \\ A_i^{ba} & A_i^{bb} & A_i^{bc} \\ A_i^{ca} & A_i^{cb} & A_i^{cc} \end{pmatrix} \tag{S2}$$

where a, b and c denote the crystallographic axes. In the paramagnetic state h_i is proportional to χ, since $\langle\mathbf{S}\rangle \sim \chi\mathbf{H}$. Compared to the case of $LiCuVO_4$ where the tensor Eq. (S2) was determined by Nawa *et al.* [14] by measurements on single crystals, $LiCuSbO_4$ is only available as powder samples. In addition, there are two different Li sites in $LiCuSbO_4$ (Fig. 1 in the main text). Li(1) is coupled most strongly to six Cu spins in three nearest neighboring chains, and Li(2) is coupled to four Cu spins in two nearest neighboring chains. The dipolar hyperfine coupling tensors for both Li sites have been calculated by lattice sum over a radius of 160 Å and are given in units [kOe/μ_B]:

$$\hat{A}_1 = \begin{pmatrix} -0.21 & 0.13 & -0.0005 \\ 0.13 & 0.27 & -1.0 \\ -0.0005 & -1.0 & -0.061 \end{pmatrix} \tag{S3}$$

$$\hat{A}_2 = \begin{pmatrix} -0.29 & 0.014 & 0.015 \\ 0.014 & -0.18 & -1.02 \\ 0.015 & -1.02 & 0.474 \end{pmatrix} \tag{S4}$$

For both Li sites, there are diagonal elements which agree with the hyperfine coupling determined from the K vs. χ plot in Fig.S1. This means, the main peak in the spectrum contains intensity of both Li sites. Note, that also the off-diagonal elements may contribute to the main peak due to the powder averaging of a specific angular dependence of h_i, and that a small transferred hyperfine coupling may exist, too.

The off-diagonal elements can transfer longitudinal electron spin fluctuations in the paramagnetic state to transverse fluctuating hyperfine fields at the Li site (cf. Eq. (1) in the main text). This would lead to spin lattice relaxation by longitudinal spin fluctuations. On the other hand, if spin correlations are peaked for certain wave vectors $\mathbf{Q}_0$ in the short-range ordered helical, SDW or nematic states, phase factors $\Theta(\mathbf{q})$ may lead to a cancellation of these off-diagonal elements making T_1^{-1} insensitive to longitudinal spin fluctuations [14]. However, such a sharp peak at $\mathbf{Q}_0$ is expected only for the field in the chain direction and not perpendicular to it due to the weak intra-chain coupling [14]. Thus, in the measurements on a powder sample of $LiCuSbO_4$ where all orientations contribute to the NMR signal one can reasonably expect that both longitudinal and transverse spin fluctuations give rise to the spin lattice relaxation at all temperatures.

Spin lattice relaxation, fits and stretched exponential relaxation: Fig.S2 shows three exemplary fits of the nuclear magnetization M versus time τ for a magnetic field of 16 T at 20 K, 4.2 K, and 1.7 K. $M(\tau)$ could then be fitted to a single exponential function $M(\tau) = M_0\{1 - f\exp[-(\tau/T_1)^b]\}$ with $f = 1$ for ideal saturation. At 4.2 K, two fits are shown: one with a fixed stretching exponent $b = 1$, and one with b as a free fitting parameter ($b = 0.81$). The difference is negligible and the extracted T_1 value is the same within the error bars. Only at the lowest temperatures a stretching exponent is necessary to fit the data, as can be seen for the data measured at 1.7 K. The lower panel of Fig. S2 shows the stretching exponent b for all fields and temperatures below 20 K. Only at higher fields > 13 T and very low T a substantial distribution of spin lattice relaxation rates appears.

Modelling of the $T_1^{-1}(T)$ dependences: Experimental $T_1^{-1}(T)$ curves for all measured magnetic fields (Fig. 5 in the main text) were modelled with Eq. (3) in the main text. The fit parameters are presented in Fig. S3.

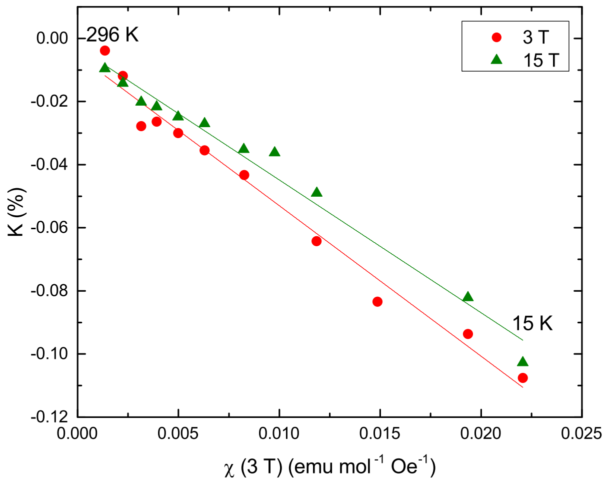

FIG. S1. Knight shift determined in 3 and 15 T versus macroscopic susceptibility of the powder sample in 3 T. The lines are fits as described in the main text.

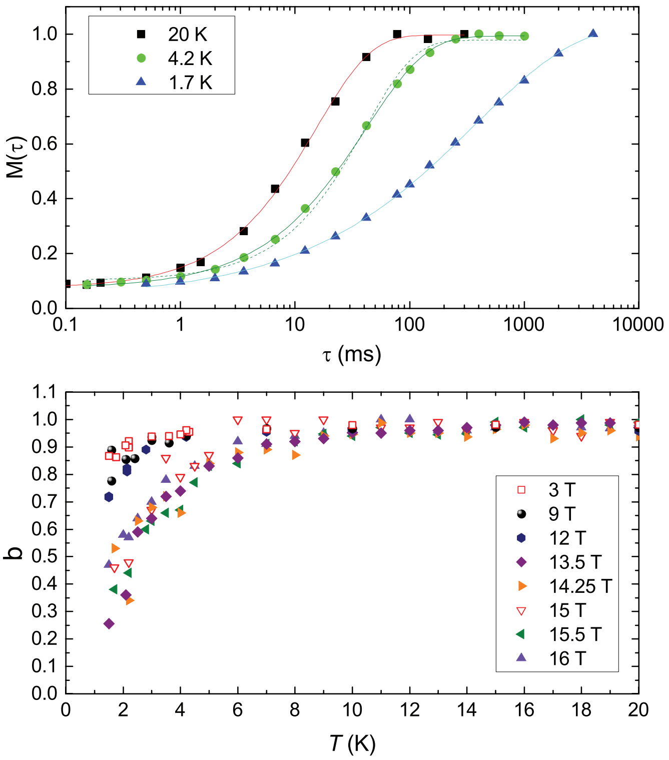

FIG. S2. Upper panel: Nuclear magnetization M versus τ in 16 T and for three different temperatures. The lines are fits as described in the text. The dashed line is a fit with b fixed to 1. Lower panel: Stretching exponent b versus temperature for all different fields.

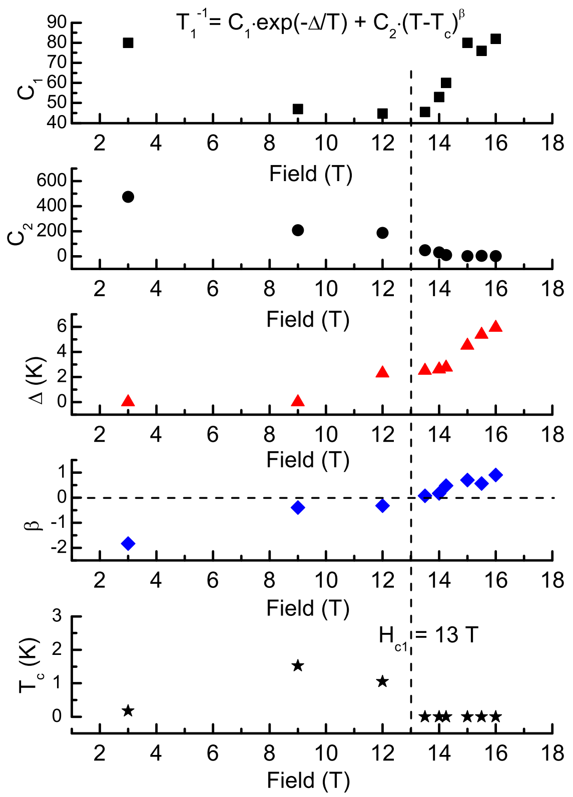

FIG. S3. Magnetic field dependence of the model parameters according to Eq. (3) in the main text. Vertical dashed line denotes the critical field $\mu_0 H_{c1} = 13\,\mathrm{T}$ corresponding to the isosbestic point in Fig. 6 in the main text.

[1] Hase, M., Terasaki, I. & Uchinokura, K. Observation of the spin-peierls transition in linear Cu^{2+} (spin-1/2) chains in an inorganic compound $CuGeO_3$. *Phys. Rev. Lett.* **70**, 3651–3654 (1993).

[2] Hase, M. *et al.* Confirmation of a one-dimensional spin-1/2 heisenberg system with ferromagnetic first-nearest-neighbor and antiferromagnetic second-nearest-neighbor interactions in $rb_2cu_2mo_3o_{12}$. *Phys. Rev. B* **70**, 104426 (2004).

[3] Agrapidis, C., Drechsler, S.-L., van den Brink, J. & Nishimoto, S. Cross-over from an incommensurate singlet spiral state with an exponentially small spin-gap to a valence bond solid state in dimerized frustrated ferromagnetic spin-chains. *arXiv:* 1612.07609v1 (2016).

[4] Nishimoto, S., Drechsler, S.-L., Kuzian, R., Richter, J. & van den Brink, J. Interplay of interchain interactions and exchange anisotropy: Stability and fragility of multipolar states in spin-1/2 quasi-one-dimensional frustrated helimagnets. *Phys. Rev. B* **92**, 214415 (2015).

[5] Dutton, S. *et al.* Quantum Spin Liquid in Frustrated One-Dimensional $LiCuSbO_4$. *Phys. Rev. Lett.* **108**, 187206 (2012).

[6] Bogdanov, A., Rößler, U., Wolf, M. & Müller, K. Magnetic structures and reorientation transitions in noncentrosymmetric uniaxial antiferromagnets. *Phys. Rev. B* **66**, 214410 (2002).

[7] Meiboom, S., Sethna, J., Anderson, P. & Brinkman, W. Theory of the blue phase of cholesteric liquid crystals. *Phys. Rev. Lett.* **46**, 1216 (1981).

[8] Wilhelm, H. *et al.* Confinement of chiral magnetic modulations in the precursor region of FeGe. *J. Phys.: Condens. Matter* **24**, 294204 (2012).

[9] Toledano, J. & Toledano, P. *The Landau theory of phase transitions* (World Scientific, 1987).

[10] Yamashita, M. First order phase transition accompanying soliton lattices near a tricritical point - chiral smectic c liquid crystal in an electric field -. *J. Phys. Soc. Jpn.* **56**, 1414 (1987).

[11] Schaub, B. & Mukamel, D. Phase diagrams of systems exhibiting incommensurate structures. *Phys. Rev. B* **32**, 6385 (1985).

[12] Kegler, C., Büttgen, N., Krug von Nidda, H.-A. & Loidl, A. NMR study of lineshifts and relaxation rates of the one-dimensional antiferromagnet $LiCuVO_4$. *Phys. Rev. B* **73**, 104418 (2006).

[13] Smerald, A. & Shannon, N. Angle-resolved nmr: Quantitative theory of ^{75}as T_1 relaxation rate in $bafe_2as_2$. *Phys. Rev. B* **84**, 184437 (2011).

[14] Nawa, K., Takigawa, M., Yoshida, M. & Yoshimura, K. Anisotropic spin fluctuations in the quasi one-dimensional frustrated magnet licuvo4. *Journal of the Physical Society of Japan* **82**, 094709 (2013).
